# Supplementary figures and images for: SARS-CoV-2 antibody magnitude and detectability are driven by disease severity, timing, and assay
Source: medRxiv. 2021 Mar 5:2021.03.03.21251639. Preprint. [Version 1] doi: 10.1101/2021.03.03.21251639 (PMC7941652; doi:10.1101/2021.03.03.21251639)

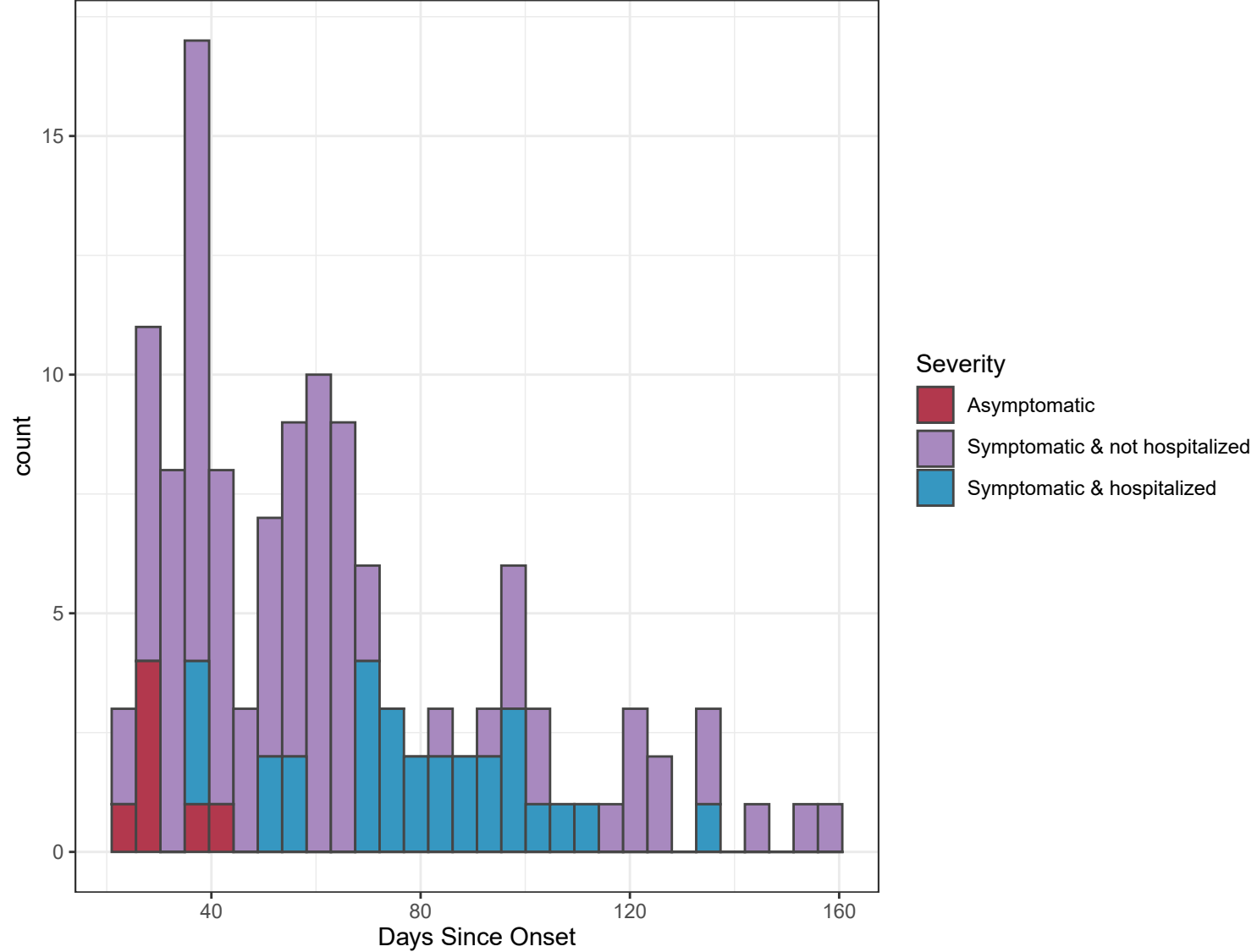

Supplement: Supplement — ary Figure 1: Distribution of time since infection at baseline visit, by severity. Days since symptom onset (if symptomatic) or positive PCR test (if asymptomatic) of the baseline visit, stratified by severity. [file media-1.pdf]

# B

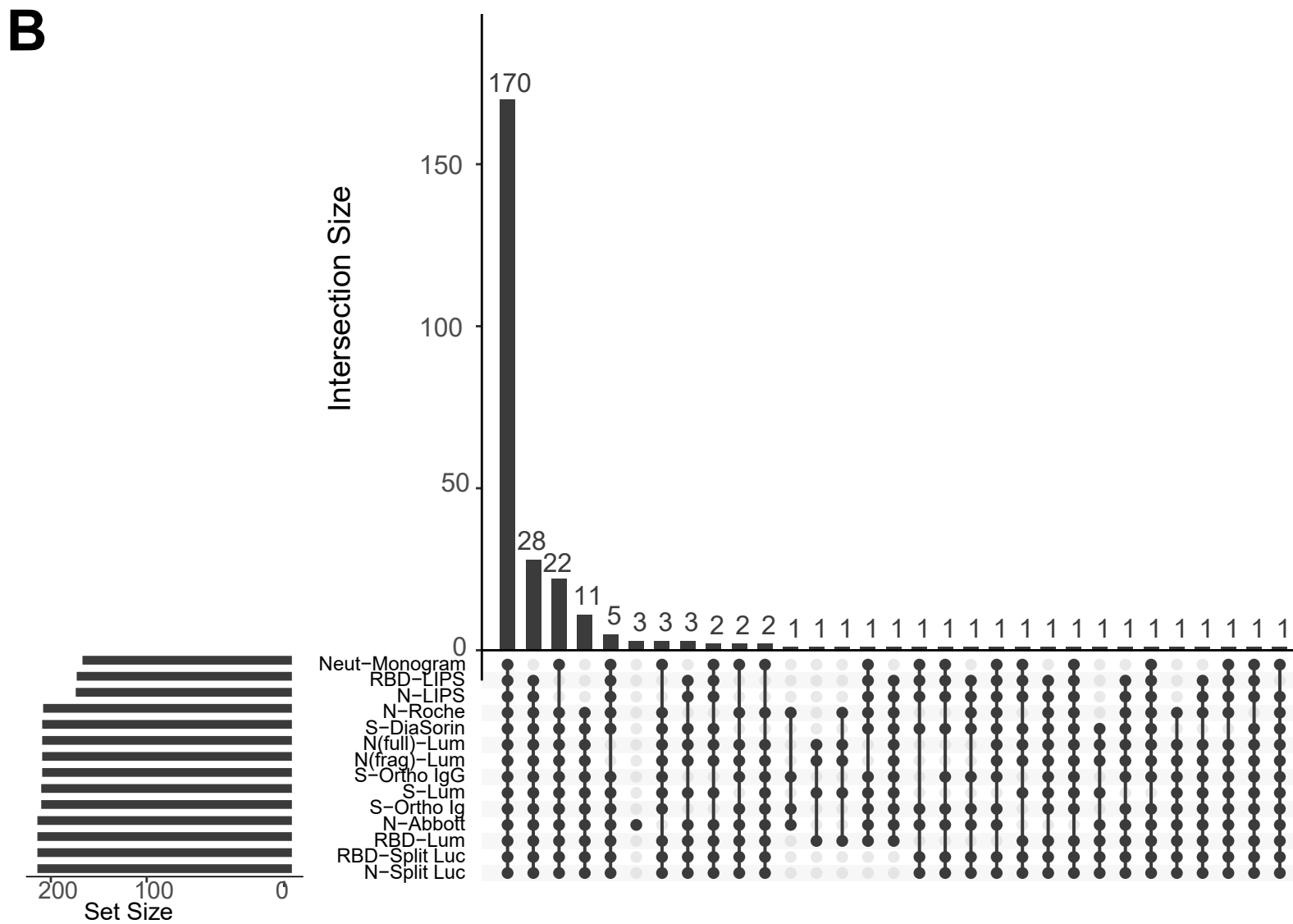

Supplement: Supplement — ary Figure 2: Sample inclusion for each platform. (A) Of the 128 total individuals included, how many individuals had at least one sample tested on each assay. (B) Of the 267 total samples tested, how many samples were tested on each assay. [file media-2.pdf]

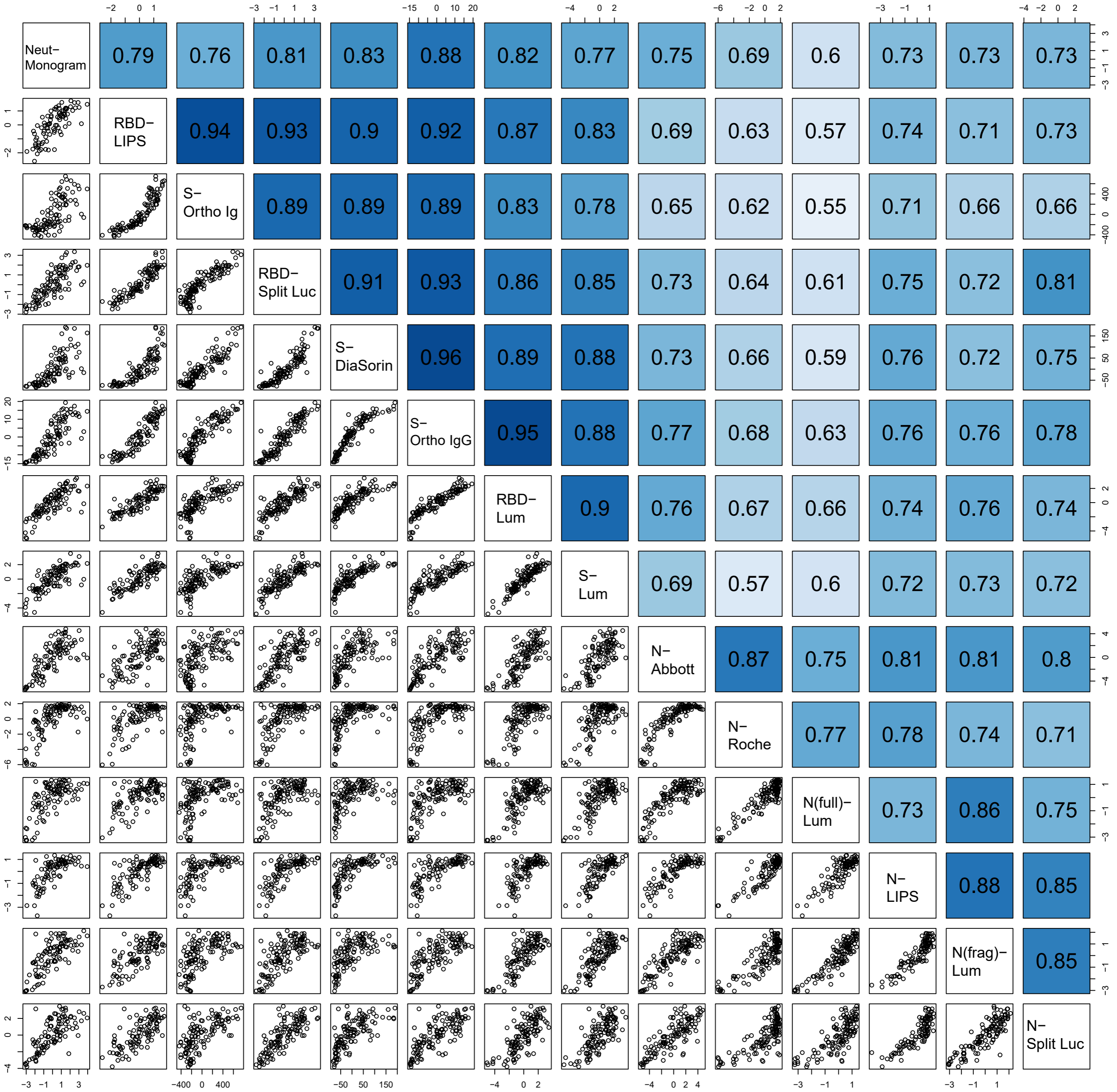

Supplement: Supplement — ary Figure 3: Correlation between all assays. The values of the (A) random intercepts, (B) all raw data, (C) all raw data from less than 90 days after symptom onset (‘early’), and (D) all raw data from greater than 90 days after symptom onset (‘late’) are shown on the lower triangle. The Spearman rank correlation coefficients and p-values of the observed coefficients are shown on the upper triangle. [file media-3.pdf]

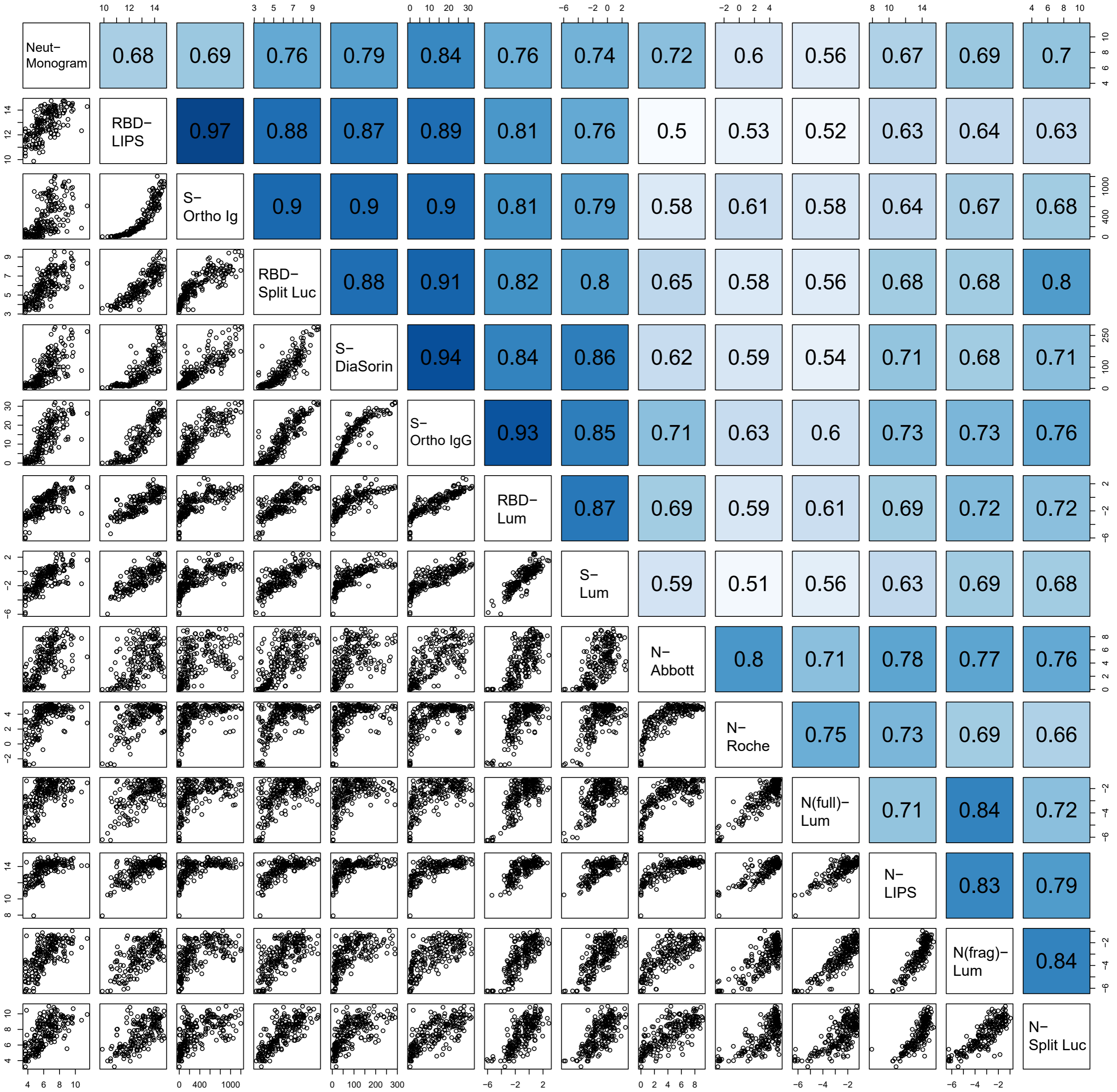

Supplement: Supplement [file media-4.pdf]

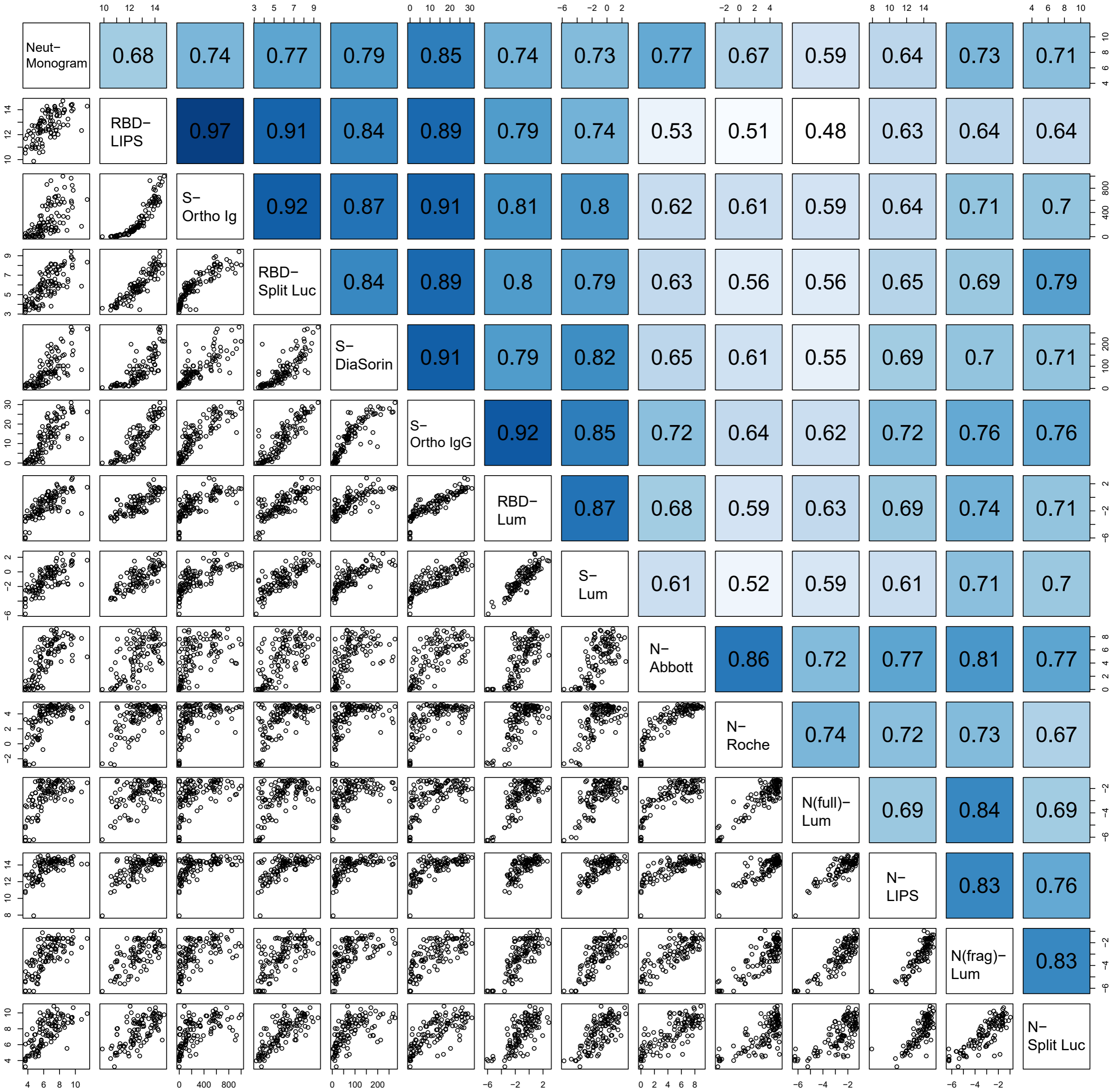

Supplement: Supplement [file media-5.pdf]

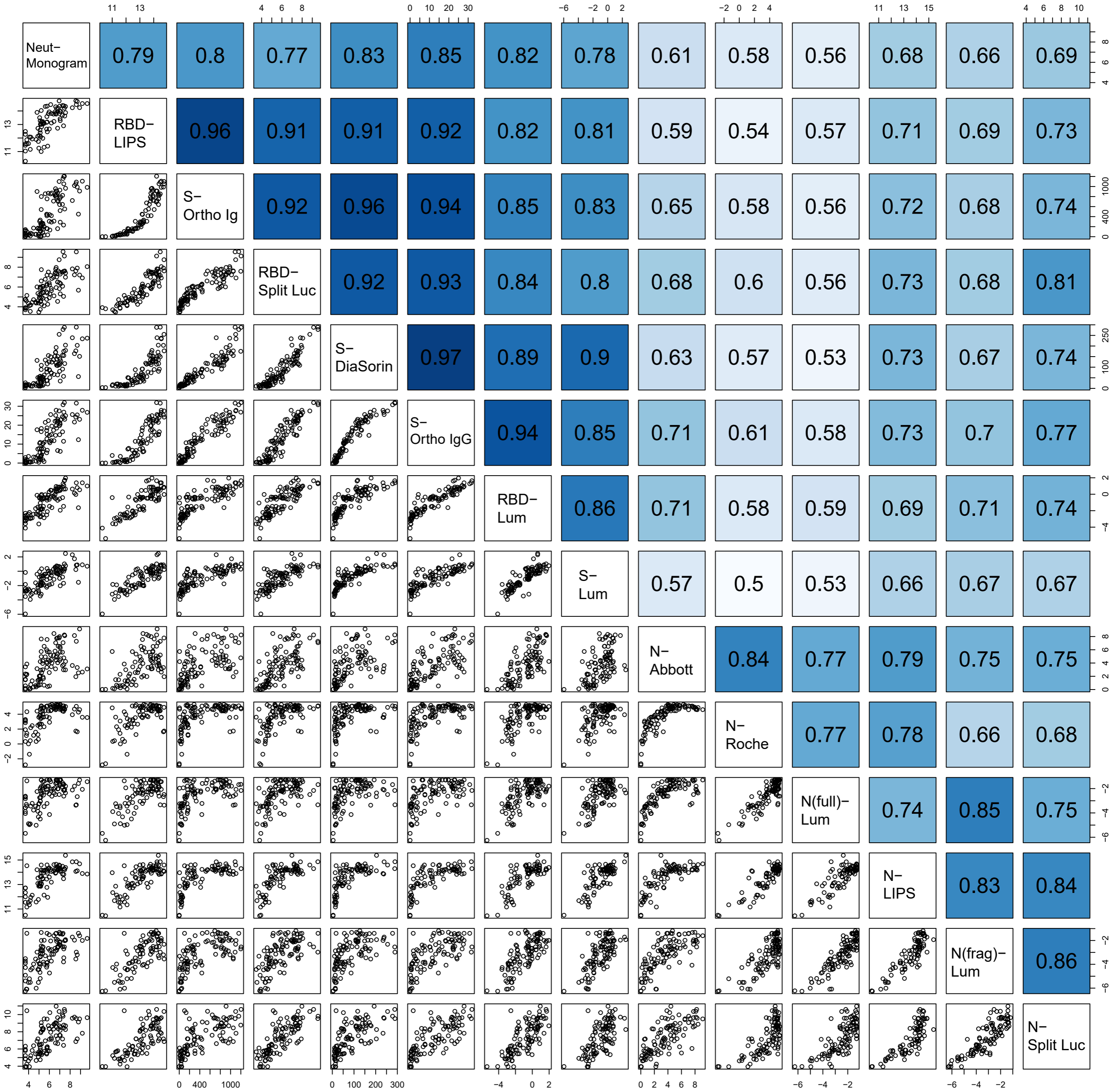

Supplement: Supplement [file media-6.pdf]

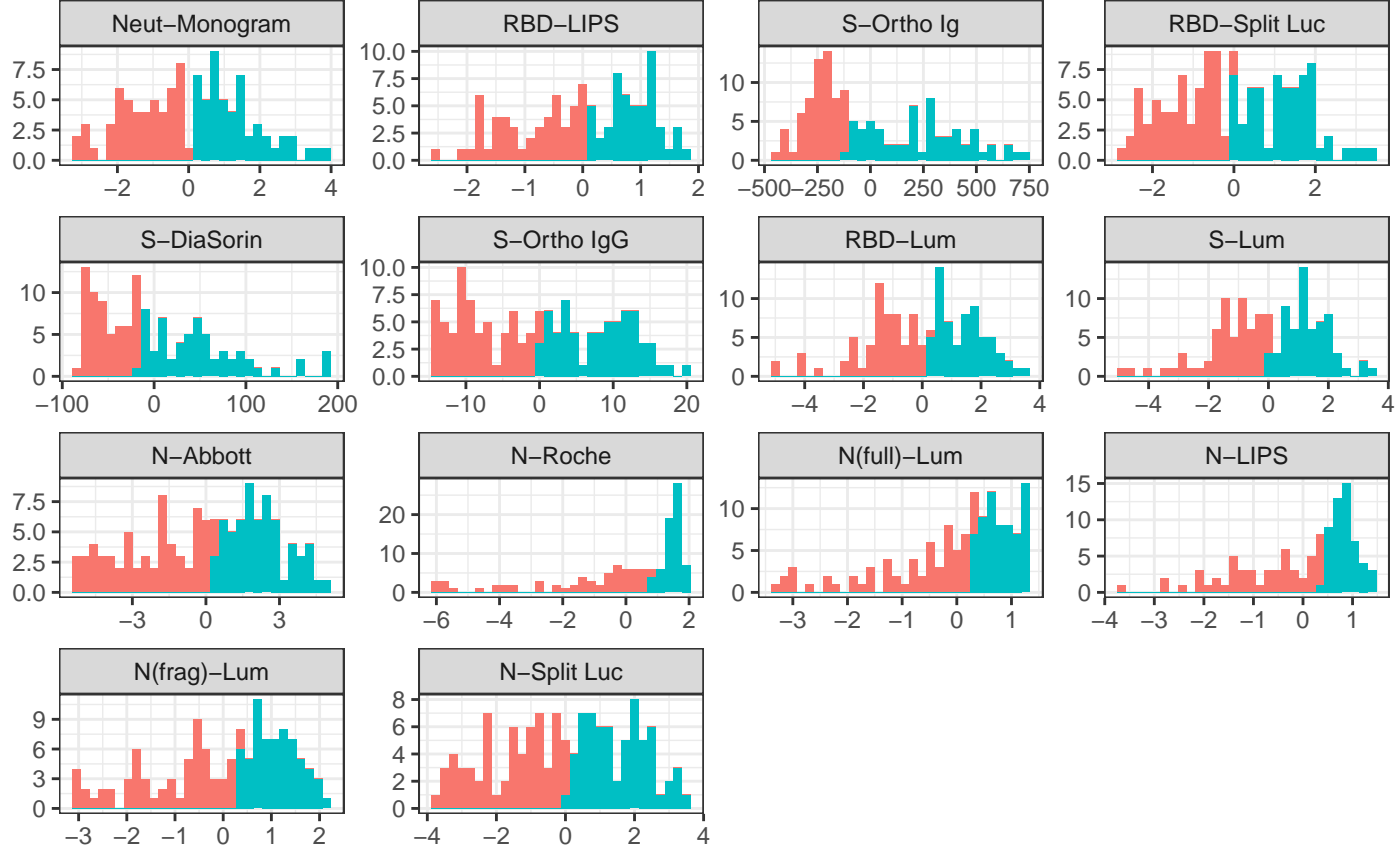

Supplement: Supplement — ary Figure 4: Distribution of the random intercept of antibody values for each individual by assay. X-axis is the raw value of the random intercept from the linear mixed effects model. Red signifies random intercept values in the bottom half of that assay, and blue signifies random intercept values in the top half of that assay. These binary values were used as outcome variables in the random forests modeling. [file media-7.pdf]

Raw data

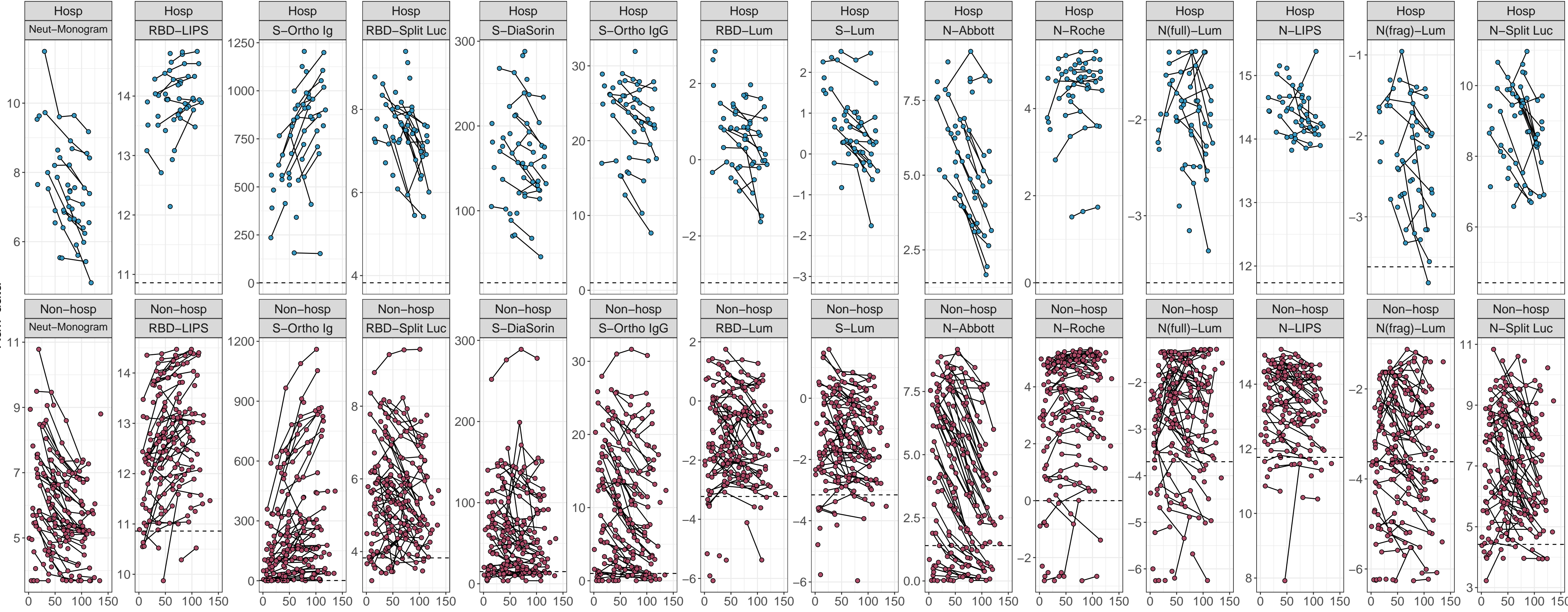

Supplement: Supplement — ary Figure 5: Raw data by time, hospitalization status, and assay. Raw antibody response data are either log-transformed or not transformed, according to Supplementary Table 1. The x-axis represents time since seroconversion in days, where seroconversion was assumed to occur (if at all) 21 days after symptom onset (if symptomatic) or 21 days after positive PCR test (if asymptomatic). The cutoff for positivity on that assay is shown by the dashed black line. [file media-8.pdf]

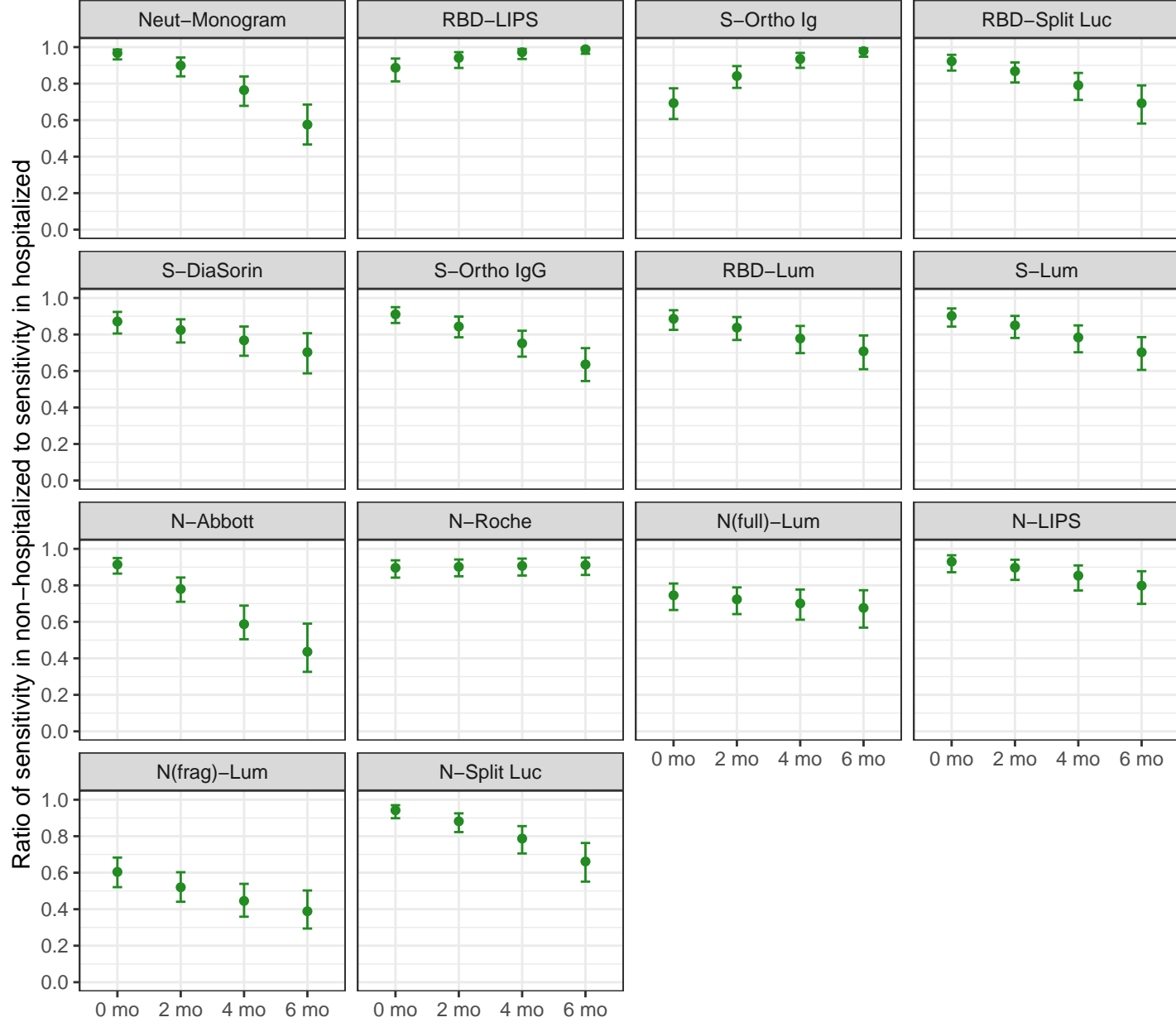

Supplement: Supplement — ary Figure 6: Ratio of sensitivity in non-hospitalized individuals to hospitalized individuals over time. Posterior median estimates and 95% credible intervals shown. [file media-9.pdf]

Negative Predictive Value

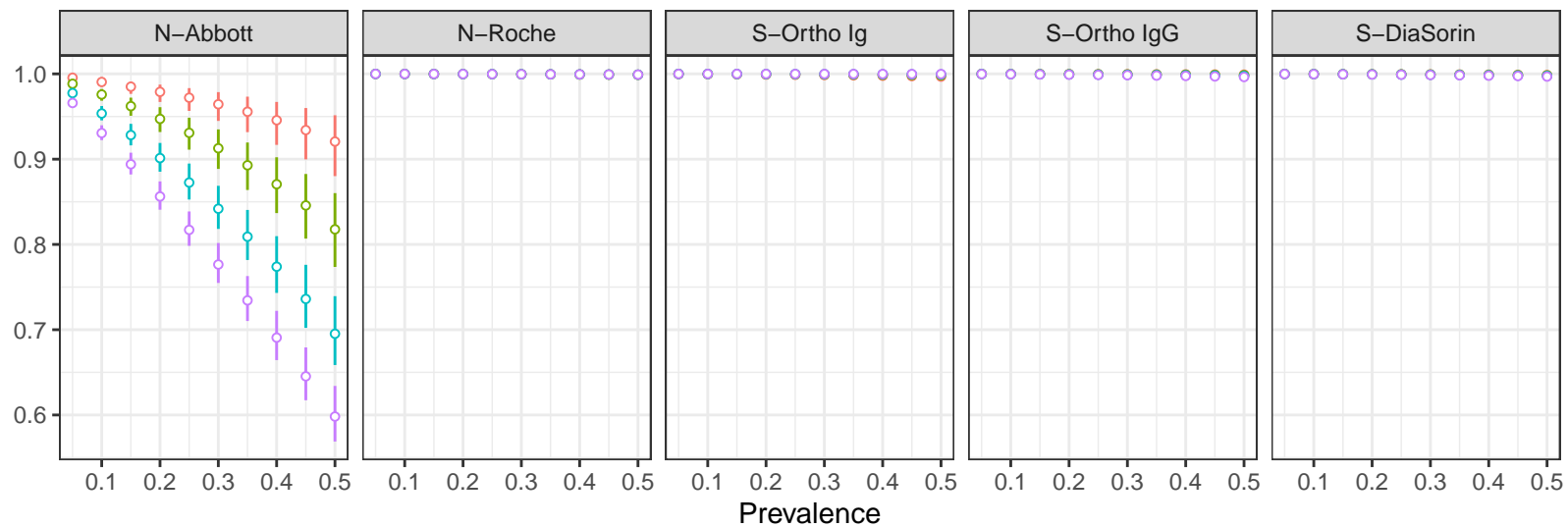

Time

- 0 months
- 2 months
- 4 months
- 6 months

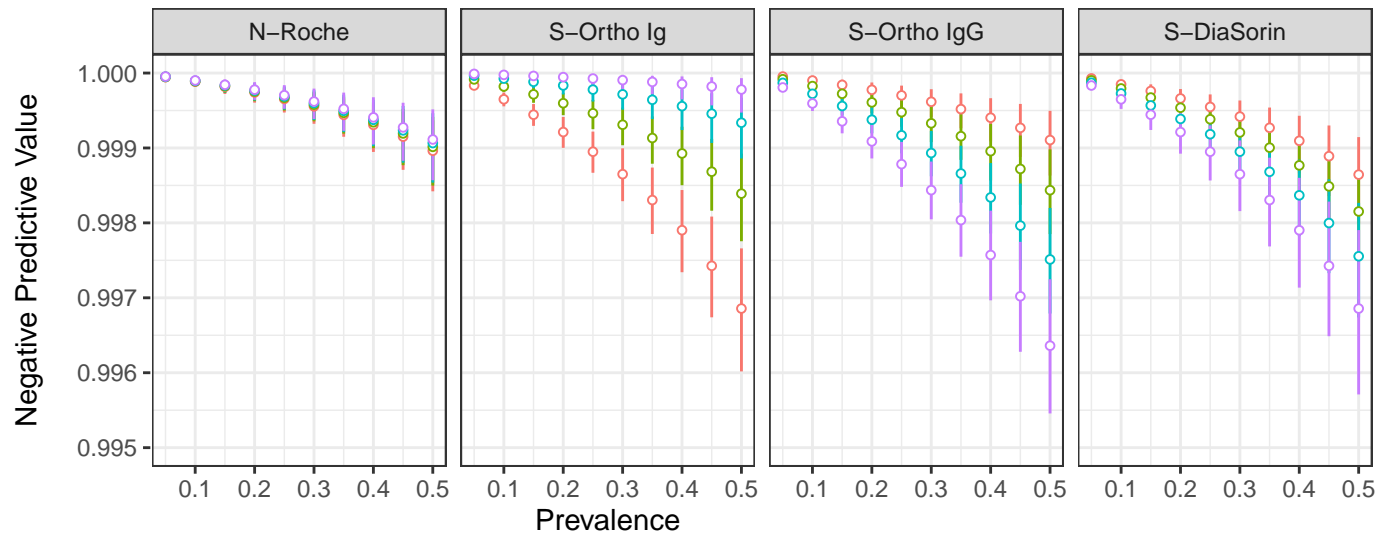

Supplement: Supplement — ary Figure 7: Negative predictive values of the commercial assays. Negative predictive values shown are based on the estimated assay sensitivities for non-hospitalized individuals in Figure 5B, for a range of prevalence between 5% and 50% (x-axis). Lower panels show the same data with a smaller range in the y axis to visualize small differences. [file media-10.pdf]
